# Magnetic Nanocomposites for the Remote Activation of Sulfate Radicals for the Removal of Rhodamine B

**DOI:** 10.3390/nano13071151

**Published:** 2023-03-23

**Authors:** Pranto Paul, Marissa Nicholson, J. Zach Hilt

**Affiliations:** Department of Chemical & Materials Engineering, University of Kentucky, Lexington, KY 40506-0046, USA

**Keywords:** magnetic nanocomposite, alternating magnetic field, organic contaminant, rhodamine B

## Abstract

The widespread presence of numerous organic contaminants in water poses a threat to the ecological environment and human health. Magnetic nanocomposites exposed to an alternating magnetic field (AMF) have a unique ability for magnetically mediated energy delivery (MagMED) resulting from the embedded magnetic nanoparticles; this localized energy delivery and associated chemical and thermal effects are a potential method for removing contaminants from water. This work developed a novel magnetic nanocomposite—a polyacrylamide-based hydrogel loaded with iron oxide nanoparticles. For this magnetic nanocomposite, persulfate activation and the contamination removal in water were investigated. Magnetic nanocomposites were exposed to AMF with a model organic contaminant, rhodamine B (RhB) dye, with or without sodium persulfate (SPS). The removal of RhB by the nanocomposite without SPS as a sorbent was found to be proportional to the concentration of magnetic nanoparticles (MNPs) in the nanocomposite. With the addition of SPS, approximately 100% of RhB was removed within 20 min. This removal was attributed primarily to the activation of sulfate radicals, triggered by MNPs, and the localized heating resulted from the MNPs when exposed to AMF. This suggests that this magnetic nanocomposite and an AMF could be a unique environmental remediation technique for hazardous contaminants.

## 1. Introduction

A large number of organic pollutants have caused serious environmental problems in recent decades. Although increasingly stringent laws and regulations have been implemented, dealing effectively with organic pollutants has continued to be a challenge and a major environmental issue [1]. Consequently, the exploration of novel and effective solutions to unnaturally high-concentration organic water pollution is always regarded as difficult. To date, numerous biological, physical, chemical, electrical, and electrochemical techniques for the removal of organic matter have been developed. From these methodologies, advanced oxidation processes (AOPs) have recently been extensively employed because of their simple, efficient, and feasible approach [2]. In recent years, a new advanced oxidation approach utilizing persulfate to create sulfate radicals (SO_4_^−^•) as an in situ chemical oxidation has gained popularity [3,4]. SO4^−^• has a high oxidation potential, comparable to that of •OH^−^ (E^0^ = 2.73 V versus NHE). Moreover, persulfate is a powerful and stable oxidizing agent (E^0^ = 2.01 V versus NHE). Persulfate can be excited to produce SO_4_^−^• by heat, transition metal ions (such as Fe^2+^, Ag^+^, or Co^2+^), alkaline conditions, and ultraviolet radiation. Among these methods, the addition of transition metal ions has been found to be a cost-effective and feasible method for activating persulfate [5].

Magnetic nanoparticles (MNPs) based on iron oxide exhibit excellent catalysis in a heterogeneous Fenton-like system, which is attributed to the presence of Fe(II) species in the magnetite structure that initiates the reaction [6]. When magnetic nanoparticles are enclosed in or coated with a non-magnetic matrix, the resulting composite is referred to as a magnetic nanocomposite; these have applications in biomedical and environmental fields [7]. Magnetic nanocomposites combine the features of a matrix or coating material with the intrinsic magnetic properties of nanoparticles, such as superparamagnetic properties. These superparamagnetic qualities enable manipulation in an external magnetic field without net remanent magnetization when the external magnetic field is removed. Additionally, the magnetic separation resulting from the MNPs is a simple, low-cost method for extracting contaminants from polluted water or slurries and is frequently more effective than procedures such as centrifugation and membrane filtration [8,9,10,11,12,13]. These distinctive properties of nanocomposites with MNPs have led to their development for environmental remediation applications. Leng et al. used polyhydroquinone-coated iron oxide to activate persulfate and then degrade RhB. In addition, they found that an increase in temperature accelerated RhB decomposition, which matched the pseudo-first-order kinetic model [5]. In a similar study, Pang et al. developed magnetic graphene oxide and used it to activate persulfate and then degrade 2,4-dichlorophenol. They reported that the synergistic effect of magnetic nanoparticles and graphene oxide enhanced persulfate activation and the radicals dispersed on the catalyst’s surface degraded the most 2,4-dichlorophenol [14].

In addition, the magnetic field-assisted approach has a substantial effect on the removal of various contaminants. By altering the parameters of the magnetic field, it is possible to tailor the contamination removal rate and selectivity [15]. Li et al. investigated the use of biochar pyrolyzed from wheat straw to remove the cationic dye methylene blue in the presence of a magnetic field (MF). Using an MF, the results demonstrated that wheat straw biochar could effectively absorb pollutants [16]. In similar study, Hao et al. found that a magnetic field improved methylene blue’s adsorption onto organo-bentonite [17].

When magnetic nanocomposites are exposed to an AMF, MNPs embedded in magnetic nanocomposites absorb energy through Néel relaxation and Brownian relaxation. This localized energy delivery and associated heating can be utilized to degrade contaminants. He et al. employed ball-milled, sulfidated microscale zero-valent iron (S-mZVIbm) particles in combination with a low-frequency AC electromagnetic field (EMF) to degrade TCE and found that electromagnetic heating dechlorinated TCE four times faster than without heating. They hypothesized that the enhanced dichlorination resulted from the increased temperature and accelerated surface layer corrosion from an AC EMF [18]. In addition, Wydra et al. reported that with AMF, MNPs in the presence of hydrogen peroxide accelerated the generation of reactive oxygen species (ROS) via Fenton-like chemistry [9]. However, there is no report on the utilization of magnetic nanocomposites and an AMF to activate persulfate and remove organic contaminants in water. With the localized heating produced by the magnetic nanocomposite when exposed to AMF, it is expected that persulfate activation could be enhanced and initiate organic pollutant removal.

In this work, magnetic nanocomposites based on polyacrylamide-based hydrogels loaded with iron oxide nanoparticles (IONP-Aam nanocomposites) were synthesized using free radical polymerization. Since rhodamine B is commonly found in industrial wastewater, it was chosen as the model contaminant to be removed in this study. In the hydrogel nanocomposite, RhB and persulfate are within the composite, and during AMF treatment, sulfate radical generation is increased at the surface of the IONPs, leading to greater removal of RhB. The sulfate radicals exhibit high reactivity and a short lifespan. Subsequent to RhB degradation, any remaining radicals likely undergo rapid conversion into sulfate ions. Here, the study successfully demonstrated: (1) the influence of MNPs and AMF strength on the sorption of RhB with IONP-Aam nanocomposites; (2) the effect of MNPs in nanocomposites and AMF on persulfate oxidation to remove RhB; and (3) a possible mechanism for the removal of RhB using AMF, persulfate, and nanocomposites.

## 2. Materials and Methods

Magnetic nanoparticles synthesis: A one-pot co-precipitation method was used to synthesize the iron oxide nanoparticles. Briefly, 40 mL aqueous solution of FeCl_3_∙6H_2_O (Thermo Fisher Scientific, Waltham, MA, USA) and FeCl_2_∙6H_2_O (Alfa Aesar, Haverhill, MA, USA) in 2:1 M ratio (2.2 g and 0.8 g, respectively) was prepared in a sealed three-neck flask. The mixture was heated to 83.5 °C with vigorous stirring (300 rpm) under an inert environment (nitrogen flow). Once the temperature reached 83 °C, 4.5 mL of NH_4_OH (VWR chemicals, Radnor, PA, USA) was added into the mixture; the temperature was increased to 85 °C and the reaction was performed for one hour at 85 °C. The particles were then magnetically decanted and washed three times with deionized (DI) water. The nanoparticles were then re-suspended in DI water and dialyzed against DI water for 24 h with periodic water changes [9].

Magnetic nanocomposite synthesis: Magnetic nanocomposite synthesis was conducted using free-radical polymerization reactions initiated by the oxidation–reduction reaction between ammonium persulfate (APS) (Sigma Aldrich, St. Louis, MO, USA) and tetramethylethylenediamine (TEMED) (Tokyo Chemical Industry, Osaka, Japan) with the addition of iron oxide nanoparticles. First, 99.5% acrylamide (in mol) (Thermo Scientific, Waltham, MA, USA) as monomer and 0.5% *N*,*N*′-methylene bis(acrylamide) (NNMBA) (in mol) (Beantown Chemical Corporation, Hudson, NH, USA) as crosslinker were added to a scintillation vial. Then 1, 3, and 5 wt% of (monomer + crosslinker) MNPs were added to the vial to vary the MNP concentration. Then, double the amount of DI water (by mass) was added to the vial and vortexed for 10 s. Then MNPs were dispersed in the mixture using ultrasonication for ten minutes (10 s sonication followed by 5 s rest until a total of ten minutes sonication). After sonication, 0.8 wt% of (monomer + crosslinker) APS were added to the mixture and vortexed for 5 s. Then 0.16 wt% of (monomer + crosslinker) TEMED were added to the mixture and vortexed for 5 s. The nanocomposites were formed in a glass plate by transferring the mixture from vial to a glass plate very quickly. Once the nanocomposite formed (within 10 min), it was transferred to a petri dish filled with DI water and was cut into 5 mm discs. The nanocomposites were washed for 6 h and then oven dried at 75 °C overnight.

Swelling study of nanocomposites: The swelling study of the nanocomposite was conducted in DI water. The nanocomposite was initially weighed and placed in a vial with 5 mL DI water and kept for 5 min at a specific temperature. The nanocomposite was then wiped with a Kimwipe and reweighed. The Q for 5 min exposure was calculated by dividing the final weight of the nanocomposite by the initial weight. The nanocomposite was placed in the same vial with DI water and exposed to the specific temperature for an additional 5 min. The nanocomposite was reweighed after wiping with Kimwipe and the Q for 10 min exposure was calculated by dividing the final weight by the initial weight of the nanocomposite. The kinetic swelling study was conducted for 90 min at different exposure temperatures.

AMF treatment and RhB detection: A Taylor Winfield magnetic induction source was used in the AMF treatment to observe the temperature profiles of the nanocomposite, using a fiber optic temperature sensor (Luxtron FOT Lab kit, LumaSense Technologies Inc., Milpitas, CA, USA). A 400 μL mixture of 0.02 mM RhB (Acros organics, Waltham, MA, USA) and 100 mg nanocomposite with or without 8 mM SPS in a 1 mL glass vial was prepared and vortexed for 10 s. The mixture was exposed for 5, 10, 15, and 20 min to the AMF. The supernatant was magnetically decanted. An additional 400 μL of DI water was added to the supernatant and vortexed for 10 s to obtain enough solution for UV–vis characterization and kept for 5 min to have the maximum mass transfer. Then, 400 μL of the solution was added to a quartz cuvette and absorption was measured at 555 nm. The experiment was conducted for varying MNP concentrations in the nanocomposite and with different AMF strengths.

Pseudo-first-order kinetic model plot: The pseudo first-order kinetic model was plotted using the following equation

ln (A_t_/A_0_) = (−k × t)

where A_t_ is the RhB absorbance in UV–vis after AMF treatment, A_0_ is the initial RhB absorbance in UV–vis, and k = reaction rate constant.

Desorption of sorbed RhB: A 400 μL mixture of 0.02 mM Rh B and 100 mg nanocomposite with or without 8 mM SPS in a 1 mL glass vial was prepared and vortexed for 10 s. The mixture was exposed to AMF coil with 34.32 kA/m strength of AMF for 15 min. Then, the supernatant was magnetically decanted; 400 μL DI water was added to the supernatant and vortexed for 10 s to obtain enough solution for UV–vis characterization and kept for 5 min for maximum mass transfer. Subsequently, 400 μL of the solution was placed in a quartz cuvette and absorption was measured at 555 nm. The remaining supernatant was removed and fresh 400 μL DI water was added to the nanocomposites and kept for 24 h at room temperature. Then, 400 μL DI water was added and kept for 5 min and then 400 μL of the solution was taken to a quartz cuvette and absorption was measured at 555 nm. The remaining supernatant was removed and fresh 400 μL DI water was added to the nanocomposites and kept for another 24 h (48 h in cumulative) at room temperature and analyzed by UV–Vis. After 24 h (72 h in total), the desorption study was conducted again.

## 3. Results and Discussion

**Figure 1:**
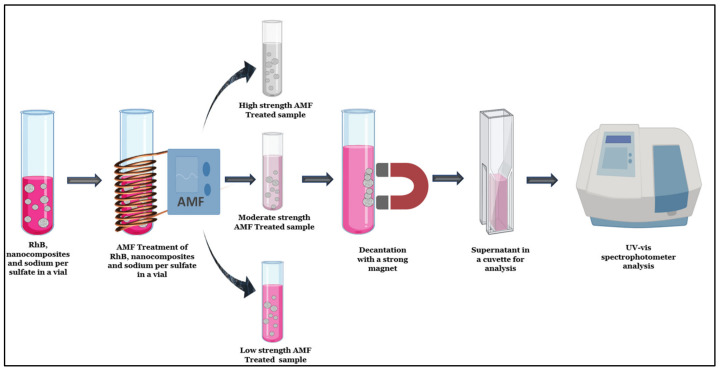
Schematic representation of the AMF treatment with magnetic nanocomposite and sodium persulfate to remove RhB. Created using biorender.com.

Here, magnetic nanocomposites using chemically initiated free radical polymerization were synthesized. The magnetic nanocomposites were characterized and utilized with persulfate to remove RhB from water under various treatment conditions. To induce the production of sulfate radicals from sodium persulfate (SPS), the magnetic nanocomposites were subjected to AMF. RhB was used as a model contaminant and exposed to the (nanocomposite, AMF, SPS) system to determine the system’s ability to remove contamination. Figure 1 illustrates the experimental method for the study of RhB removal.

**Figure 2:**
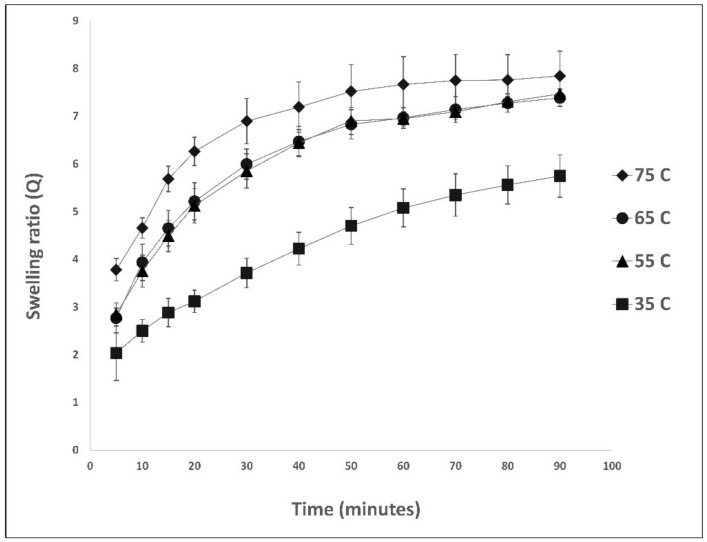
Kinetic swelling study of IONP-acrylamide nanocomposite exposed to different temperatures. (N = 3, error bars represent the standard deviation).

The magnetic nanocomposites were characterized through a kinetic swelling study, as summarized in Figure 2. By mimicking the RhB removal experiment with varying AMF strength, a swelling study was conducted in a water bath using 3% MNP in nanocomposite at the same stable temperatures of the varying AMF strength. When subjected to greater temperatures, magnetic nanocomposites swell significantly more. This is attributed to the fact that an increase in temperature causes the dissociation of hydrogen bonds in an intra-polymer complex, resulting in an increase in the number of hydrophilic sites capable of interacting with water molecules and a decrease in the crosslinking density of the hydrogel. This extends the distance between the polymeric chains, thereby facilitating the diffusion of water into the nanocomposites [19,20]. The swelling continues until equilibrium is reached across all temperature exposures.

To determine the removal of RhB from water via sorption or degradation, RhB and nanocomposite were mixed and exposed to AMF with or without SPS. To observe the removal of RhB following the AMF treatment, the supernatant was examined using a UV–vis spectrophotometer.

**Figure 3:**
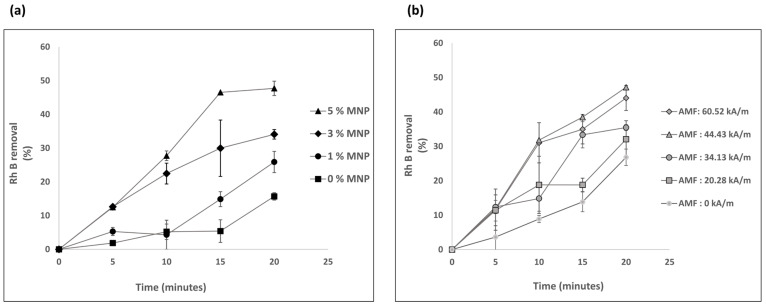
Removal of RhB without SPS (**a**) at different MNP concentrations in the nanocomposites in an AMF strength of 34.13 kA/m; (**b**) at different strengths of AMF with a nanocomposite of 3% MNP. (N = 3, error bars represent the standard deviation).

The removal of RhB with IONP-Aam nanocomposites without SPS for different MNP concentrations in nanocomposites and AMF strengths is based on sorption and presented in Figure 3. The sorption of RhB in the nanocomposite increases with time in various concentrations of MNP in nanocomposites and with different AMF strengths. Figure 3a indicates that MNPs in nanocomposites aid in the sorption of RhB. Without MNPs in the nanocomposite, the highest removal of RhB in 20 min is around 15%. With an increase in MNPs from 0% to 5% in the nanocomposite, sorption increased by approximately 30% in 20 min. This increased sorption in the higher MNP concentrations in nanocomposite is the result of additional sorption sites created by the MNPs. In contrast, increasing the AMF strength from 20.28 kA/m to 60.52 kA/m did not have a significant impact on sorption (Figure 3b). At the lowest AMF exposure strength for 20 min, the nanocomposite containing 3% MNP removed approximately 30% of RhB. With a threefold increase in AMF strength, RhB elimination increases by approximately 15%. Due to only sorption occurring, the RhB removal without SPS did not fit the kinetic model.

**Figure 4:**
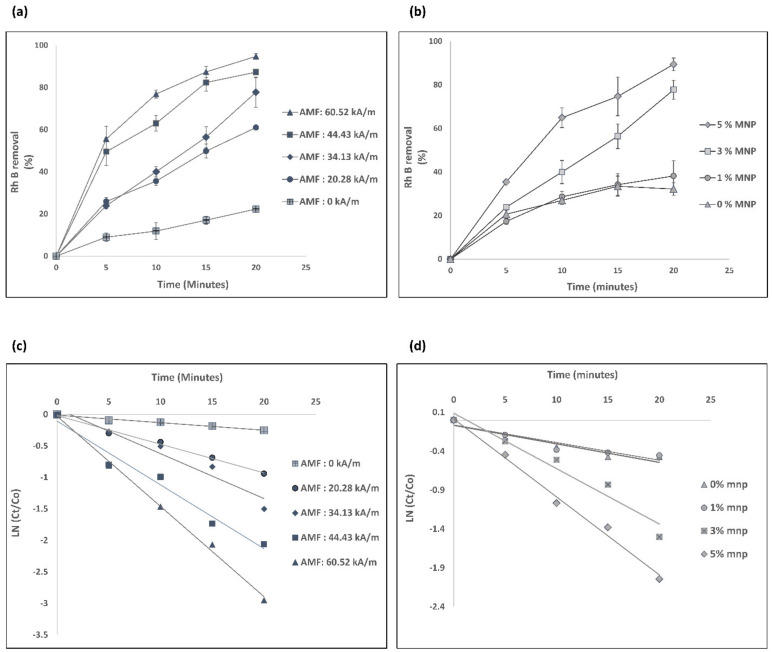
Removal of RhB with SPS (**a**) at different strengths of AMF with a nanocomposite of 3% MNP; (**b**) with different MNP concentrations in nanocomposites with an AMF strength of 34.13 kA/m. Pseudo-first-order plot of the RhB degradation (**c**) at different strengths of AMF with a nanocomposite of 3% MNP; (**d**) at different MNP concentrations in the nanocomposite with an AMF strength of 34.13 kA/m; (N = 3, error bars represent the standard deviation).

The removal of RhB increased significantly when 8 mM SPS is added, as shown in Figure 4a,b. To confirm the degradation of RhB both in water and within nanocomposites in the presence of SPS, desorption experiments were conducted with and without SPS, as demonstrated in Figure S1. It was found that after desorption for 72 h, the sample with SPS desorbed 3.8% of the removed RhB and the sample without SPS desorbed 66% of the removed RhB. This indicates that both in the water and within the nanocomposites, RhB was degraded in the presence of SPS and the removal of RhB in the presence of SPS is dominated by degradation. This could be attributed to the enhanced sulfate radical activation by the magnetic nanocomposites when exposed to an AMF.

The localized heating generated in the magnetic nanocomposite was controlled by the AMF strength and MNP concentration variation in nanocomposites, as shown in Figure S2; by controlling these variables, RhB can be degraded to various degrees, up to approximately 100% with the persulfate activation within 20 min (Figure 4). Figure 4a demonstrates that the removal of RhB with nanocomposite containing 3% MNP and 8 mM SPS for 20 min at 0 kA/m AMF strength was approximately 20%. By increasing the AMF strength to 60.52 kA/m under the same conditions, approximately 100% of RhB was removed. Figure 4b illustrates that when the proportion of MNPs in the nanocomposite was increased from 0% to 5%, the percentage of RhB removal increased to around 100% from 30%.

The degradation of RhB in the presence of SPS and nanocomposites over time was also plotted in a pseudo-first-order kinetic model, and it fitted well, with a R2 > 0.95 with varying AMF strength and R2 > 0.90 with varying MNP concentrated nanocomposites. The reaction rate constants of RhB degradation in presence of SPS with different AMF strength and MNP concentration can be calculated with this pseudo-first-order kinetic model. The calculated reaction rates are summarized in Table 1 and Table 2. Reaction rate increases with both increased AMF strength and increased MNP concentration in the nanocomposites.

By studying RhB degradation at various steady-state temperatures, depending on AMF strength and MNP concentration, an Arrhenius-type relationship was also determined. On the basis of the reaction rates observed in Table 1 and Table 2, it was found that the reaction rate depends on the temperature resulting from the AMF strength and nanocomposite’s MNP content. To find the activation energy of the corresponding reaction, the Arrhenius equation was used.

**Figure 5:**
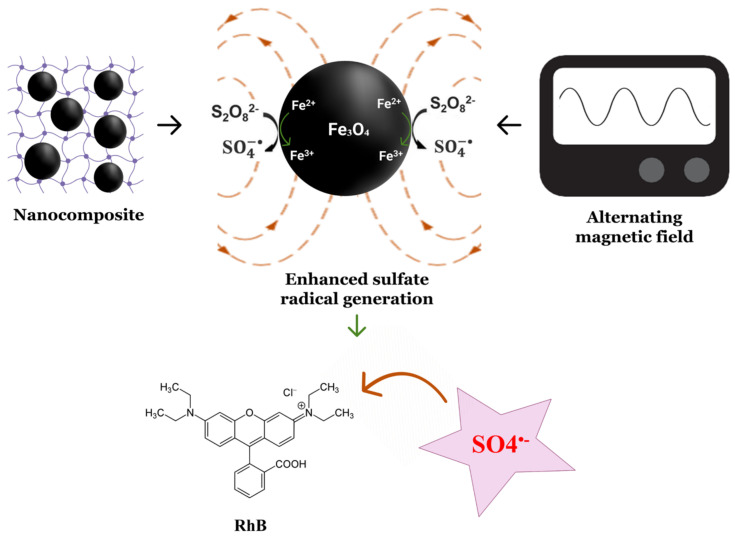
Potential mechanism for the production of enhanced sulfate radicals for RhB degradation. Created using biorender.com.

The Arrhenius equation is:

k = A exp(−Ea/RT)

where A is the pre-exponential factor, Ea is the activation energy, R is the ideal gas constant, and T is the reaction temperature. The linear relationship between ln(k) and 1/T is plotted in Figure S3. The derived activation energy and the pre-exponential factor are listed in Table 1 and Table 2. The activation energy was determined to be 24.3 KJ mol^−1^ and 25.3 KJ mol^−1^, respectively, for varying AMF strengths and MNP concentrations in the nanocomposite. It is lower than for the other Fe-based catalyst reported in earlier studies for RhB degradation via the production of sulfate radicals, proving the advantage of IONP-Aam nanocomposites [1,5]. A significant enhancement of SPS decomposition and sulfate radical generation also occurs with the temperature increase [21]. When subjected to AMF, the surface temperature of MNPs in nanocomposites is higher than the temperature of the solution. When SPS comes into contact with this high-temperature MNP surface through sorption, SPS decomposition is initiated and more radicals are produced. This increased catalytic activity of the MNP surface under exposure to AMF is likely the primary cause of accelerating RhB degradation and decreasing activation energy. Figure 5 demonstrates the mechanism of enhanced sulfate radical generation and RhB attack by these radicals.

## 4. Conclusions

Here, IONP-Aam nanocomposite was synthesized using free radical polymerization and the nanocomposites were subjected to AMF with SPS for enhanced sulfate radical production to remove a model organic contaminant named RhB. The sorption capability of nanocomposites without SPS to remove RhB was also investigated, and it was found that MNPs in nanocomposites impacted RhB sorption, although AMF strength variation had no significant effect on sorption. Through localized heating of MNPs in nanocomposite in AMF exposure, persulfate activation was initiated significantly, and RhB was degraded nearly completely. The RhB degradation rates with these nanocomposites and AMF system were found to be comparable to other RhB degradation methods [1,5]. The degradation with varying amount of MNPs in the nanocomposites and with varying AMF strengths were found to follow a pseudo-first-order kinetic model. Overall, this study demonstrates a novel method to enhance the oxidation of persulfate via localized heating driven by magnetic nanocomposite and AMF, to efficiently degrade RhB. Therefore, the suggested technique has promising potential for the degradation of printing and dyeing wastewater, as well as other refractory organic pollutants, such as trichloroethylene/tetrachloroethylene.

## Figures and Tables

**Table 1:** Reaction rate constant of RhB degradation at different AMF strengths with a nanocomposite of 3% MNP and their corresponding activation energy and Arrhenius constant.

AMF Strength Variation with Nanocomposite of 3% MNP and SPS				
AMF Strength (kA/m)	S.S. Temperature (K)	Rate Constant (min^−1^)	E (kJ·mol^−1^)	A (min^−1^)
0	298	0.012	24.3	585
20.28	308	0.045		
34.13	328	0.071		
44.43	338	0.101		
60.52	348	0.143		

**Table 2:** Reaction rate constant of RhB degradation at different MNP concentrations in nanocomposites with an AMF strength of 34.13 kA/m.

MNP Concentration Variation in Nanocomposite with SPS in 34.13 kA/m AMF Strength				
MNP Concentration (%)	S.S. Temperature (K)	Rate Constant (min^−1^)	E (kJ·mol^−1^)	A (min^−1^)
0	293	0.023	25.3	672
1	303	0.024		
3	328	0.071		
5	348	0.100		

## Data Availability

The data presented in this study are available on request from the corresponding author.

## Supplementary Materials

The following supporting information can be downloaded at: https://www.mdpi.com/article/10.3390/nano13071151/s1, Figure S1: (a) Removal of RhB with 3% MNP in magnetic nanocomposites exposed to 34.32 kA/m AMF strength for 15 min with and without SPS. (b) Cumulative desorption of sorbed RhB in DI water at room temperature after AMF exposure. Figure S2: Heating profile of IONP-acrylamide magnetic nanocomposites exposed to AMF. (a) For 3% MNPs in varying AMF strengths; (b) for 34.13 kA/m strength AMF with varying MNP concentrations in nanocomposites. The temperature profile of 0 kA/m exposure and 0% MNP nanocomposite exposed to 34.13 kA/m respectively in (a) and (b) is decreasing with time due to the AMF chiller. This demonstrates that the heating in these two samples is insignificant compared to the chilling by AMF chiller. Figure S3: Arrhenius plots derived from pseudo-first-order kinetic model from RhB degradation at different MNP concentrations in nanocomposites with an AMF strength of 34.13 kA/m and at different strengths of AMF with a nanocomposite of 3% MNP.
