# Supplementary material for: Magnetic Nanocomposites for the Remote Activation of Sulfate Radicals for the Removal of Rhodamine B
Source: Nanomaterials (Basel). 2023 Mar 23;13(7):1151. doi: 10.3390/nano13071151 (PMC10097114; doi:10.3390/nano13071151)
Supplement: Supplementary file 1 [file nanomaterials-13-01151-s001.zip › nanomaterials-2264030-supplementary.pdf]

Supplementary Materials

Article

Magnetic Nanocomposites for the Remote Activation of Sulfate Radicals for the Removal of Rhodamine B

Pranto Paul, Marissa Nicholson and J. Zach Hilt *

Department of Chemical & Materials Engineering, University of Kentucky, Lexington, KY 40506-0046, USA
* Correspondence: zach.hilt@uky.edu; Tel.: +1-859-257-9844

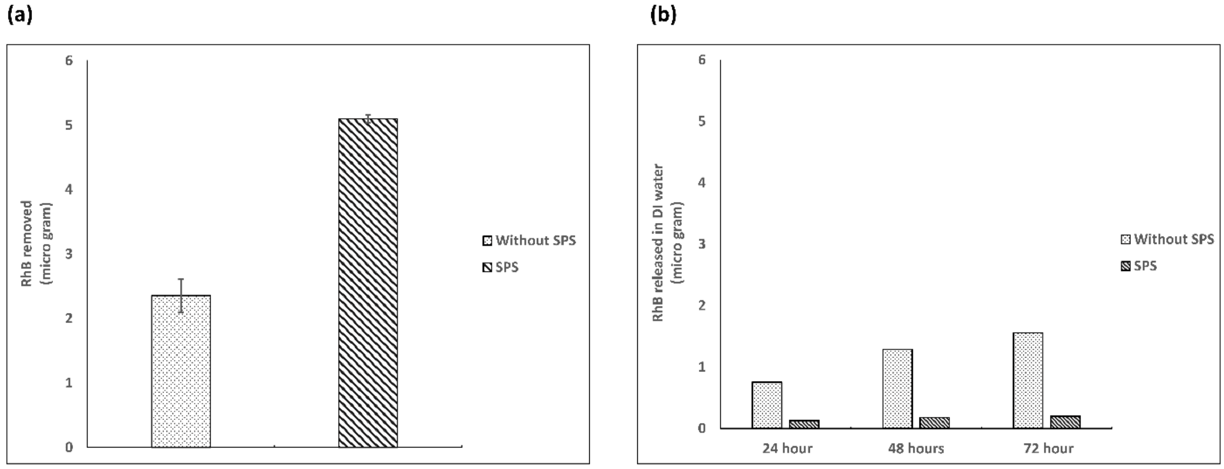

Figure S1. (a) Removal of RhB with 3% MNP in magnetic nanocomposites with exposure to 34.32 kA/m AMF strength for 15 min with and without SPS. (b) Cumulative desorption of sorbed RhB in DI water at room temperature after AMF exposure.

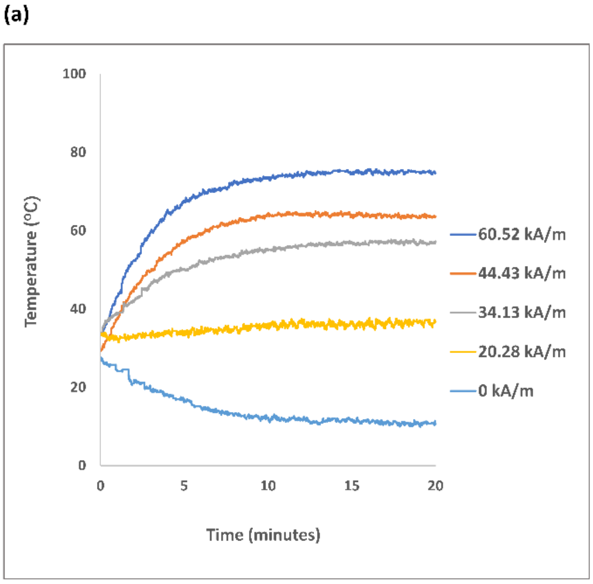

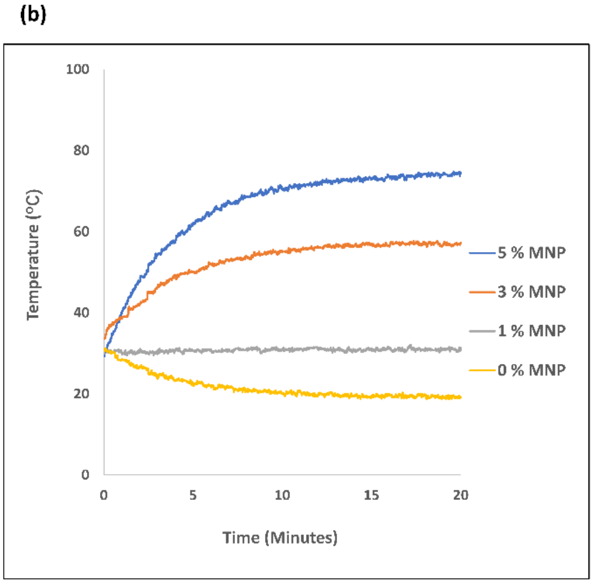

Figure S2: Heating profile of IONP-acrylamide magnetic nanocomposites exposed to AMF. (a) For 3% MNPs in varying AMF strength; (b) For 34.13 kA/m strength AMF with varying MNP concentration in nanocomposite. The temperature profile of 0 kA/m exposure and 0% MNP concentrated nanocomposite exposed to 34.13 kA/m respectively in (a) and (b) is decreasing with time due to the AMF chiller. This demonstrates that the heating in these two samples is insignificant compared to the chilling by AMF chiller. .

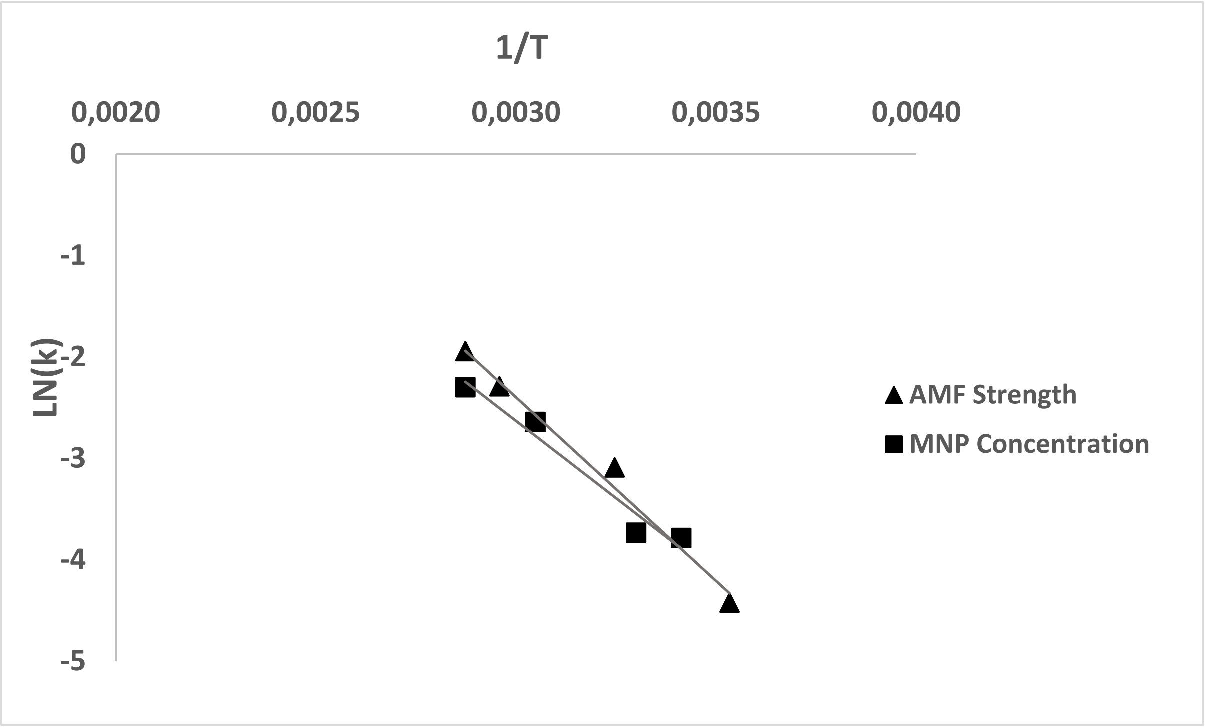

Figure S3. Arrhenius plots derived from pseudo first order kinetic model from RhB degradation at different MNP concentration in nanocomposite in an AMF strength of 34.13 kA/m and at different strength of AMF with a nanocomposite of 3 % MNP.
